# The insect, *Galleria mellonella*, is a compatible model for evaluating the toxicology of okadaic acid

**DOI:** 10.1007/s10565-018-09448-2

**Published:** 2018-11-13

**Authors:** Christopher J. Coates, Jenson Lim, Katie Harman, Andrew F. Rowley, David J. Griffiths, Helena Emery, Will Layton

**Affiliations:** 1 Department of Biosciences, College of Science, Swansea University, Swansea, Wales SA2 8PP UK; 2 Biological and Environmental Sciences, Faculty of Natural Sciences, University of Stirling, Stirling, Scotland FK9 4LA UK

**Keywords:** Haemocytes, Innate immunity, Oxidative stress, Phenoloxidase, Shellfish-poisoning syndrome, Immunotoxicology

## Abstract

The polyether toxin, okadaic acid, causes diarrhetic shellfish poisoning in humans. Despite extensive research into its cellular targets using rodent models, we know little about its putative effect(s) on innate immunity. We inoculated larvae of the greater wax moth, *Galleria mellonella,* with physiologically relevant doses of okadaic acid by direct injection into the haemocoel (body cavity) and/or gavage (force-feeding). We monitored larval survival and employed a range of cellular and biochemical assays to assess the potential harmful effects of okadaic acid. Okadaic acid at concentrations ≥ 75 ng/larva (≥ 242 μg/kg) led to significant reductions in larval survival (> 65%) and circulating haemocyte (blood cell) numbers (> 50%) within 24 h post-inoculation. In the haemolymph, okadaic acid reduced haemocyte viability and increased phenoloxidase activities. In the midgut, okadaic acid induced oxidative damage as determined by increases in superoxide dismutase activity and levels of malondialdehyde (i.e. lipid peroxidation). Our observations of insect larvae correspond broadly to data published using rodent models of shellfish-poisoning toxidrome, including complementary LD_50_ values: 206–242 μg/kg in mice, ~ 239 μg/kg in *G*. *mellonella*. These data support the use of this insect as a surrogate model for the investigation of marine toxins, which offers distinct ethical and financial incentives.

**Electronic supplementary material:**

The online version of this article (10.1007/s10565-018-09448-2) contains supplementary material, which is available to authorized users.

## Introduction

Diarrhetic shellfish poisoning (DSP) is one of several recognised shellfish-poisoning syndromes including amnesic, neurotoxic, and paralytic. Upon consumption of bivalves, crustaceans, sea urchins, and finfish contaminated with the polyether toxin, okadaic acid (C_44_H_68_O_13_), DSP manifests as nausea, vomiting, diarrhoea, abdominal cramps, and chills (Vale and Sampayo 2002; Valdiglesias et al. 2013). These symptoms tend to develop within 4 h of intoxication and can last for several days. Okadaic acid (OA) and its derivatives (DTXs 1–3) are produced mainly by the dinoflagellate genera, *Prorocentrum* and *Dinophysis*, and < 50 μg per kg contaminated shellfish tissue can induce symptoms of DSP in humans (Dominguez et al. 2010). In addition to being a potent inhibitor of serine-threonine phosphatases, OA is a tumour promoter with genotoxic and neurotoxic properties (Munday 2013). The occurrences of harmful algal blooms and the incidences of human intoxication from contaminated shellfish are predicted to increase annually due to climate change (Gobler et al. 2017). This represents a significant health concern for the public and additional financial burden for the aquaculture and fishing industries (Gobler et al. 2017).

The coastal waters surrounding the UK are considered a potential hot spot for *Dinophysis* species blooms and the concomitant release of DSP-associated toxins. Moreover, between 1999 and 2009, DSP blighted UK shellfish harvesters with 19 outbreaks recorded—the most of any shellfish-poisoning toxin (Hinder et al. 2011). That said, the acute or chronic impacts of lipophilic phytotoxins, including pectenotoxins and azaspiracids, on immune functionality are poorly understood. The traditional method for screening contaminated shellfish was the mouse bioassay (Yasumoto et al. 1985), but this has been replaced within the last decade by analytical methods. Such approaches are limited to screening only and do not offer insight into the mechanism(s) of action of OA. Therefore, we require a reliable in vivo model to explore in-depth the effects of OA at cellular and molecular levels.

Larvae of the greater wax moth, *Galleria mellonella*, have been developed to interrogate the pathogenicity of microbes (Lim et al. 2018), efficacy of novel biopharmaceuticals, and toxicity of diverse chemicals (Allegra et al. 2018; Maguire et al. 2016; Wuensch et al. 2018). In addition to their ease-of-use, low maintenance costs, and lack of legislative/ethical restrictions, the recent publication of the *G*. *mellonella* genome by Lange et al. (2018) should see their popularity increase as an alternative model to rodents. A biological advantage of using larvae is that the insect innate immune system is mechanistically similar to aspects of vertebrate innate immunity, e.g. antimicrobial peptide production, pathogen recognition via ligand interactions, and phagocyte-mediated respiratory burst (Bergin et al. 2005; Browne et al. 2013; Butt et al. 2016). We consider the applicability of *G*. *mellonella* larvae as an in vivo system can extend to screening the harmful effects of known, and emerging, marine toxins that confront commercially important shellfish and humans.

The overall aim of our study was to carry out an assessment on the relative toxicity of OA in an alternative model. To address this, we prospected the larvae of *G*. *mellonella* to compare (1) LD_50_ values in insects with those available from rodents, (2) two inoculation methodologies (intrahaemocoelic injection versus force-feeding), and (3) the cellular and biochemical responses to physiologically relevant doses of OA (25–125 ng/larva = 80.65–403.25 μg/kg). We arrived at this concentration range by selecting values above and below the upper regulatory limit set by the Food Standards Agency UK, which is 160 μg OA per kg contaminated shellfish tissue (FSA 2018).

## Materials and methods

### Reagents

Unless stated otherwise, all reagents used in this study were of the highest purity available when purchased from Sigma-Aldrich (Dorset, UK). Certified okadaic acid (OA; C_44_H_68_O_13_) was sourced from TOCRIS Biosciences (UK; Cat. No. 1136).

### Insects

Final instar larvae of the greater wax moth, *Galleria mellonella*, were sourced from Livefoods Direct (Sheffield, UK) and Pets at Home Ltd. (Cheshire, UK) or reared in-house following the guidelines proposed by Jorjão et al. (2018). Each insect was inspected for damage/infection and stored subsequently for no more than 7 days at 15 °C in wood shavings provided by the commercial suppliers. Only healthy larvae weighing 0.31 ± 0.04 g were used. Larvae were assigned randomly to each treatment and all experiments were performed on at least three independent occasions.

### Survival studies (LD_50_)

Insects were inoculated via intrahaemocoelic injection (26/27-gauge needle) through the last left pro-leg with 20 μL of increasing concentrations of OA: 25, 50, 75, 100, and 125 ng per larva. Stock concentrations of OA were prepared in dimethyl sulfoxide (DMSO, Cat. No. D8418) and diluted to working concentrations in (0.2 μm) filter-sterilised phosphate-buffered saline (PBS, Cat No. P4417) pH 7.4 prior to injection. Negative controls consisted of PBS only (20 μL) or PBS containing 5% DMSO (20 μL). Post-inoculation, larvae were placed in 90-mm petri dishes containing Whatman filter paper and wood shavings and incubated at 30 °C in the dark. Survival was recorded over a 96-h period.

As OA exposure is via the ingestion of contaminated shellfish tissues, we compared inoculation methods. In a second experiment, insect larvae (reared in-house) were either injected (described above) or force-fed (gavage) OA at 25, 75, and 125 ng/larva. On each occasion, it was administered using a 27-gauge hypodermic needle and survival was recorded over a 72-h period.

### Cellular analyses of larvae injected with OA

#### Total haemocyte counts

Approximately 50 μL of haemolymph was extracted from challenged (OA) and control (PBS) larvae by piercing the insect integument with a 26/27-gauge hypodermic needle and pooling samples from two individuals at each time point (4, 24, and 48 h). Haemolymph was collected into pre-chilled (sterile) microcentrifuge tubes, diluted into PBS pH 7.4, and enumerated using an improved Neubauer haemocytometer.

#### Cytological staining

Extracted haemolymph (10 μL) was mixed immediately in a ratio of 1:1 with PBS pH 7.4 containing 0.2% trypan blue (*w*/*v*, Cat. No. T6146) for determining cellular viability. Samples were incubated for no more than 3 min at room temperature prior to counting. Trypan blue-positive (non-viable) versus un-stained (viable) haemocytes were used to calculate percentage mortality.

Phenoloxidase (PO)-positive haemocytes were identified using a staining method adapted from Aladaileh et al. (2007). Briefly, haemolymph (10 μL) was mixed with an equal volume of PBS containing 5 mM MBTH (3-methyl-2-benothiazolinone hydrazine, Cat. No. 129739), 4 mM dopamine hydrochloride (Cat. No. H8502), 4-HA (methoxyphenol, Cat. No. 54050), and 20 μg mL^−1^ lipopolysaccharides from *Escherichia coli* (Cat. No. L2630). Samples were incubated for 30 min at room temperature before being examined under bright-field optics. Haemocytes staining positively for PO appeared reddish brown in colour.

All haemocyte observations were performed in duplicate (two technical replicates per biological replicate). Fields of view were selected randomly until 200 to 300 cells were counted per sample.

### Biochemical analyses of insects inoculated with OA

All assays were performed in a SPECTROstar Nano using either the cuvette port (1 mL volume) or 96-well plate reader (~ 250 μL volume). In each case, protein concentrations of cell-free haemolymph and midgut homogenates were determined using the Biuret method with bovine serum albumin (Cat. No. A2153) as a standard.

#### Phenoloxidase activity in insects after OA injection or gavage

Haemolymph samples were removed from control (PBS) and OA-treated larvae (over a 48-h period) and centrifuged at 1000×*g* for 2 min to remove haemocytes. Supernatants were retained and placed on ice. Background absorbance readings of each reaction mixture (in the absence of haemolymph) were subtracted from final readings. Each enzyme assay (1 mL) was carried out at 28 °C in PBS pH 7.4 containing 2 mM dopamine hydrochloride (substrate) and cell-free haemolymph diluted 1:100 (*v*/*v*). PO activity was calculated by monitoring an increase in absorbance at 475 nm due to the formation of product (i.e. dopaminechrome) over a 10-min period. One unit of PO activity is defined as the increase in absorbance at 475 nm per minute per mg protein.

#### REDOX-associated activity in the insect midgut after force-feeding OA

Six insect larvae per treatment per time point were chilled on ice for 5 min and dissected to remove the midgut. These samples were pooled to represent a single biological replicate. Dissections were performed in filter-sterilised PBS pH 7.4 and homogenised using 0.1 g midgut tissues in 1 mL PBS or malondialdehyde (MDA) lysis solution. Homogenates were centrifuged at 13,000×*g* for 10 min at 4 °C to remove cellular debris prior to freezing the supernatants at − 80 °C.

#### Superoxide dismutase activity

Activity of the enzymatic antioxidant, superoxide dismutase (SOD) (EC 1.15.1.1), was detected spectrophotometrically at 560 nm using a colorimetric kit (Cat. No. ab65354) purchased from Abcam (Cambridge, UK). Water-soluble tetrazolium salts (WST-1) produce a formazan dye when reduced by superoxide anions (O_2_^**−**^). This rate of reduction shares a linear relationship with the activity of xanthine oxidase, which is inhibited by SOD. Therefore, SOD activity was calculated based on its ability to inhibit the reduction of WST-1. Assays consisted of 20 μL midgut homogenate, 20 μL enzyme working solution (xanthine oxidase), and 200 μL WST working solution (following supplier’s guidelines). Sample mixtures were incubated at 28 °C for 20 min prior to data collection. One unit of SOD activity is expressed as the increase in absorbance at 560 nm per minute per mg protein.

#### Lipid peroxidation—malondialdehyde accumulation

Oxidative deterioration of lipids was detected spectrophotometrically using a colorimetric kit (Cat. No. ab118970) from Abcam (Cambridge, UK). Briefly, midgut samples washed with PBS pH 7.4 were processed in MDA lysis solution. Sample homogenates (~ 200 μL) were added to 600 μL thiobarbituric acid reagent and incubated at 95 °C for 1 h prior to data collection. Lipid peroxidation was recorded as the increase in absorbance at 532 nm per minute per mg protein. Absorbance values were converted to concentrations (nmol) of MDA using the molar coefficient 1.56 × 10^5^ M^−1^ cm^−1^ and confirmed using the standard curve recommended by the supplier.

### Data handling

All survival experiments consisted of a minimum 30 individuals per treatment (10 larvae per independent replicate). Results from survival experiments (*n* = 30–70 per treatment, 300–490 in total) were analysed in GraphPad Prism 7.0 using the log-rank (Mantel-Cox) test (curve comparisons). We further assessed insect survival between the two inoculation methods using a three-way ANOVA: OA doses [25, 75, 125 ng], time [24, 72 h], inoculation [injection/gavage]. LD_50_ values for OA were calculated at 24 h post-inoculation by fitting data to a dose-response curve [Log10(dose)] with four parameters (min, max, EC_50_, slope of the curve). Cellular and biochemical properties of the insects challenged with okadaic acid were analysed (*n* = 18–54, 108–270 in total) using two-way ANOVA with Tukey’s multiple comparison tests. Differences were considered significant when *P* ≤ 0.05.

## Results

### Relative toxicity of OA in insect larvae

**Fig. 1:**
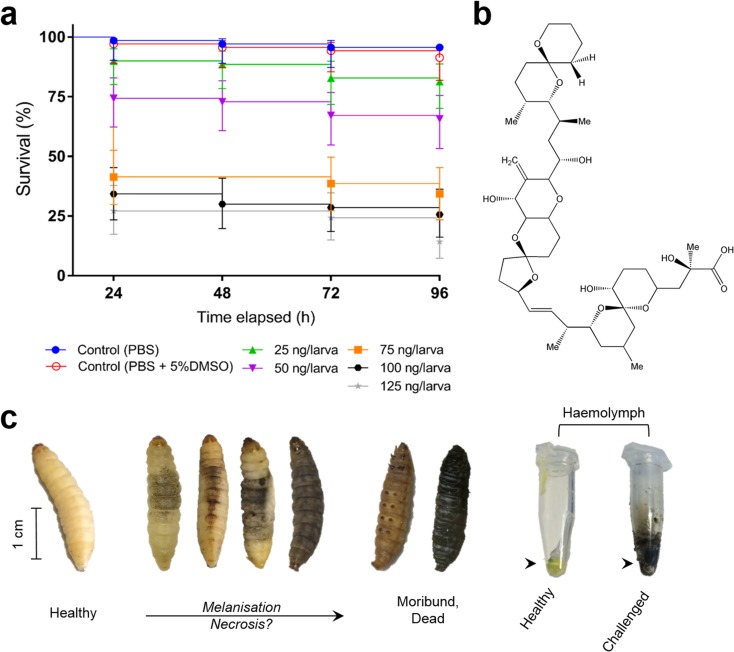
**a** Survival of *Galleria mellonella* larvae following intrahaemocoelic injection of okadaic acid, 0–125 ng/larva. Post-injection of the biotoxin, larvae were incubated at 30 °C in the dark and monitored over a 96-h period. Larvae unresponsive to touch were considered dead. Negative control groups consisted of larvae treated with 20 μL phosphate-buffered saline (PBS) or 20 μL PBS containing 5% dimethyl sulfoxide (DMSO). All values are represented by the mean with 95% CI (*n* = 70 per treatment, 490 in total). **b** Chemical structure of okadaic acid (C_44_H_68_O_13_) from the dinoflagellate *Prorocentrum concavum*. **c** Images depicting varied melanisation reactions and haemolymph discolouration in healthy (yellow appearance) and challenged larvae

Intrahaemocoelic injection of OA had a significant negative effect on the survival of *G*. *mellonella* larvae (log-rank (Mantel-Cox) curve comparison test: *χ*^2^(6) = 208.2, *P* < 0.0001; Fig. 1a, b). As the dose of OA increased, we observed more pronounced melanisation/darkening of the insect cuticle and discolouration was also reflected in the haemolymph (Fig. 1c). Analysis of the survival curves revealed no significant differences (log-rank (Mantel-Cox), *χ*^2^(1) = 1.04, *P* = 0.3078) between PBS and PBS + 5%DMSO over the 96-h period. As the toxin was lethal within 24 h post-inoculation using the concentration range of 25–125 ng/larva (equivalent to 80.65–403.25 μg [okadaic acid] per kg [tissue]), we calculated an LD_50_ value of 239.55 μg/kg [*R*^2^ = 0.951] at this time point (Fig. 1a). This falls within the range of LD_50_ values calculated for OA administered via intraperitoneal injection in the mouse bioassay (Table 1). Survival across all treatments was 100% up to 4 h post-injection.

**Table 1:** Okadaic acid administration, lethality, and pathobiology in animal models

Model system	Administration	Lethality	Pathobiology	Reference
Insect larvae [*Galleria mellonella*]	Gavage (force-feeding)	LD_50_ = 248.3 μg/kg^a^ [*R*^2^ = 0.947, *n* = 30]	Oxidative damage in the midgut—increased SOD activity and levels of MDA	This study
Insect larvae [*Galleria mellonella*]	Intrahaemocoelic injection	LD_50_ = 239.55 μg/kg^b^ [*R*^2^ = 0.951, *n* = 70]; LD_50_ = 252.8 μg/kg^a^ [*R*^2^ = 0.900, *n* = 30]	Cytotoxicity—decreased haemocyte numbers corresponding to increased levels of cell death	This study
Mouse bioassay (unknown strain)	Intraperitoneal injection	LD_50_ = 206–229 μg/kg	Small intestine and liver damage, cyanosis	Dickey et al. (1990); Tubaro et al. (2003); Aune et al. (2007)
Mice: A/J [inbred strain], BALB/c [inbred strain], C3H/He [inbred strain], C57BL/6 [inbred strain], DBA/2 [inbred strain], ICR [non-inbred]	Intraperitoneal injection	Median lethal dose 216–242.4 μg/kg	Not available	Suzuki (2012)
Mice: ICR [non-inbred], NMRI [inbred strain], Swiss mice	Gavage	Median lethal dose 300–880 μg/kg	Mild to severe damages to epithelial villi in the duodenum, jejunum, and caecum; bleeding and oedema in the lung; apoptosis in the kidney and liver	Ito et al. (2002); Le Hégarat et al. (2006); Aune et al. (2012)
Mouse (unknown strain)	Dermal application	Dose 80 ng	Severe irritation and tumour promotion	Fujiki et al. (1987)
Rat (unknown strain)	Intrahippocampal injection	Dose 7–70 ng/day	Neuronal cell death, memory loss, lipid peroxidation, protein carbonylation	He et al. (2001); Zhang and Simpkins (2010)
Rat [Wistar rats]	Intravenous (tail) injection	Dose 0.05–0.5 μg/g body weight	Blood congestion in the liver	Berven et al. (2001)
Rat [Nude rats]	Gavage (intragastric intubation)	Dose 1–4 μg/g body weight	Damages to the small intestine, shedding of epithelial cells, villus fragmentation	Berven et al. (2001)

*MDA*, malondialdehyde; *SOD*, superoxide dismutase

^a^Insects reared in-house

^b^Insects sourced commercially

Doses of OA in excess of 50 ng/larva reduced survival substantially from 74% to 41–27% (75–125 ng/larva) at 24 h. Hazard ratios (Mantel-Haenszel) of 3.11 (95% CI, 1.73–5.62), 4.34 (95% CI, 2.41–7.80), and 6.48 (95% CI, 3.63–11.55) were calculated for the increases in OA from 50 to 75, 100, and 125 ng/larva, respectively (see Supplementary Table 1 for all pairwise comparisons).

### Effect of OA on cellular and biochemical properties of *Galleria mellonella*

**Fig. 2:**
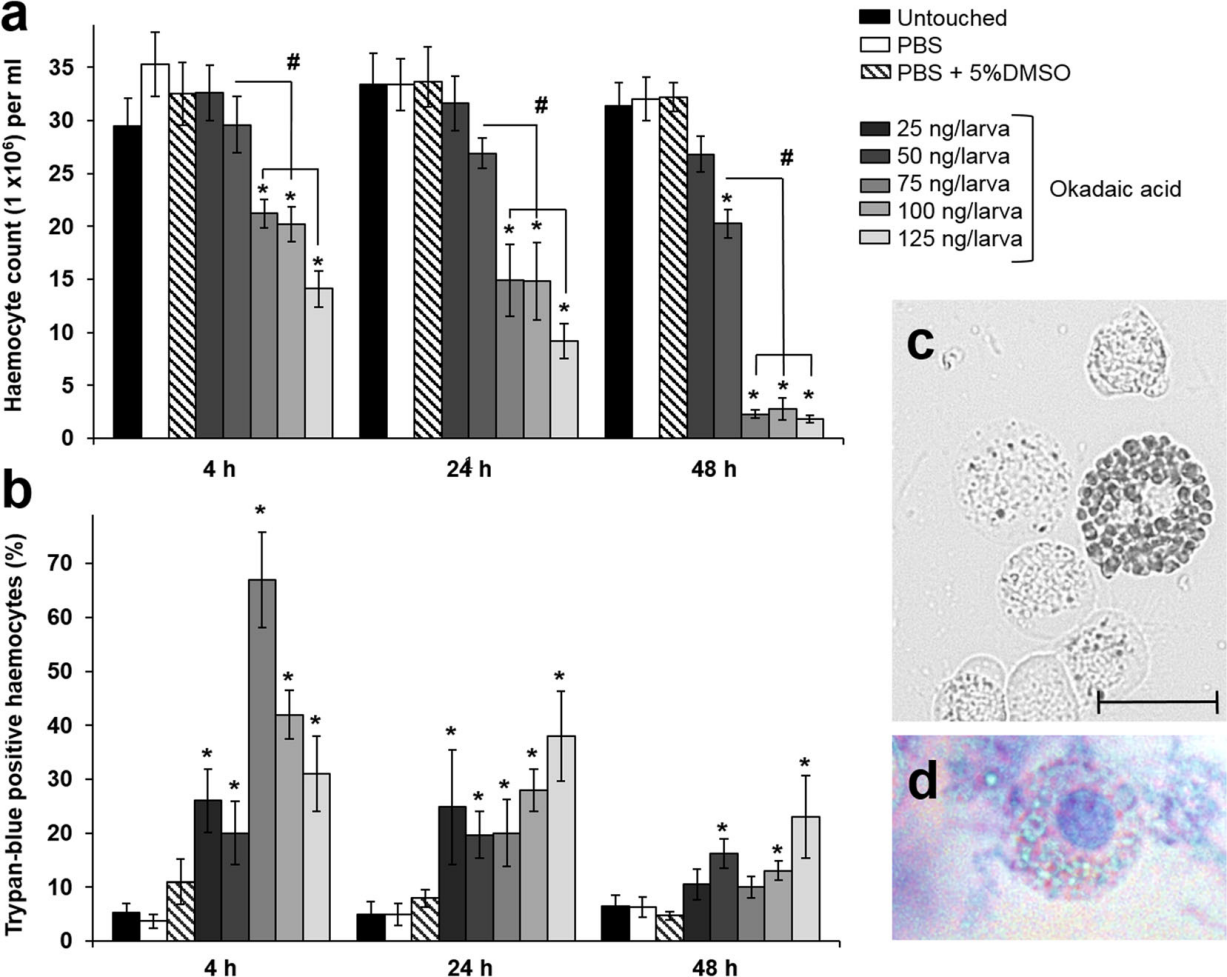
Cellular properties of *Galleria mellonella* injected with increasing concentrations of okadaic acid, 0–125 ng/larva. **a** Total haemocyte counts and **b** haemocyte viability using trypan blue exclusion were recorded over a 48-h period. All values are represented by the mean ± SE (*n* = 18 per treatment, 144 in total). An asterisk indicates a significant difference (*P* < 0.05) in haemocyte numbers relative to the PBS control. A hashtag indicates significant differences between 50 ng/larva and 75–125 ng/larva (determined by Tukey’s multiple comparisons). **c** Image representing free-floating haemocytes. Scale bar represents 10 μm. **d** Dead (or dying) haemocytes stain blue

Changes in haemocyte numbers within insects can be used to gauge the immune-suppressive or immune-stimulatory effects of diverse compounds, including toxins (Mowlds et al. 2010; Fallon et al. 2011; Browne et al. 2013; Champion et al. 2016). When injected directly into the haemocoel, OA led to significant reductions in circulating haemocyte numbers in a time (*F*(2, 116) = 21.19, *P* < 0.0001) and dose (*F*(7, 116) = 47.31, *P* < 0.0001) dependent manner (Fig. 2a). Within 4 h p.i., haemocyte numbers decreased from 3.5 × 10^7^ mL^−1^ in the PBS control to 2.1–1.4 × 10^7^ mL^−1^ in treatments ≥ 75 ng/larva. Haemocyte numbers continued to fall in treatments ≥ 75 ng/larva with 2.8–1.8 × 10^6^ mL^−1^ remaining by 48 h. Overall, time accounted for ~ 9% of the variation within the data, whereas dosage accounted for 70.4%. Haemocyte numbers within the control larvae remained unchanged for the duration of the experiment, ranging from ~ 3 to 3.5 × 10^7^ mL^−1^ (Fig. 2a).

Using the trypan blue exclusion method, we determined OA to be cytotoxic to insect immune cells (Fig. 2b). Changes observed in trypan blue-positive (i.e. dead) haemocytes also occurred in a time (*F*(2, 48) = 59.82, *P* < 0.0001) and dose (*F*(7, 48) = 51.71, *P* < 0.0001) dependent manner (Fig. 2b–d). After the initial peak in cell death at 4 h p.i. wherein 31 to 68% of haemocytes (0.4 to 1.4 × 10^7^ mL^−1^) were affected in larvae treated with 75–125 ng OA, trypan blue-positive haemocyte levels fluctuated subsequently between 10.5 and 38%. Haemocyte deaths in control larvae did not drop below 4.8% or exceed 11% over the 48-h experimental period.

Not only were there significantly fewer haemocytes overall within the haemolymph of OA-treated larvae (as low as 1.8 × 10^6^ mL^−1^; Fig. 2a), but large proportions of them (up to 23%) were dead at 48 h p.i. (Fig. 2b).

**Fig. 3:**
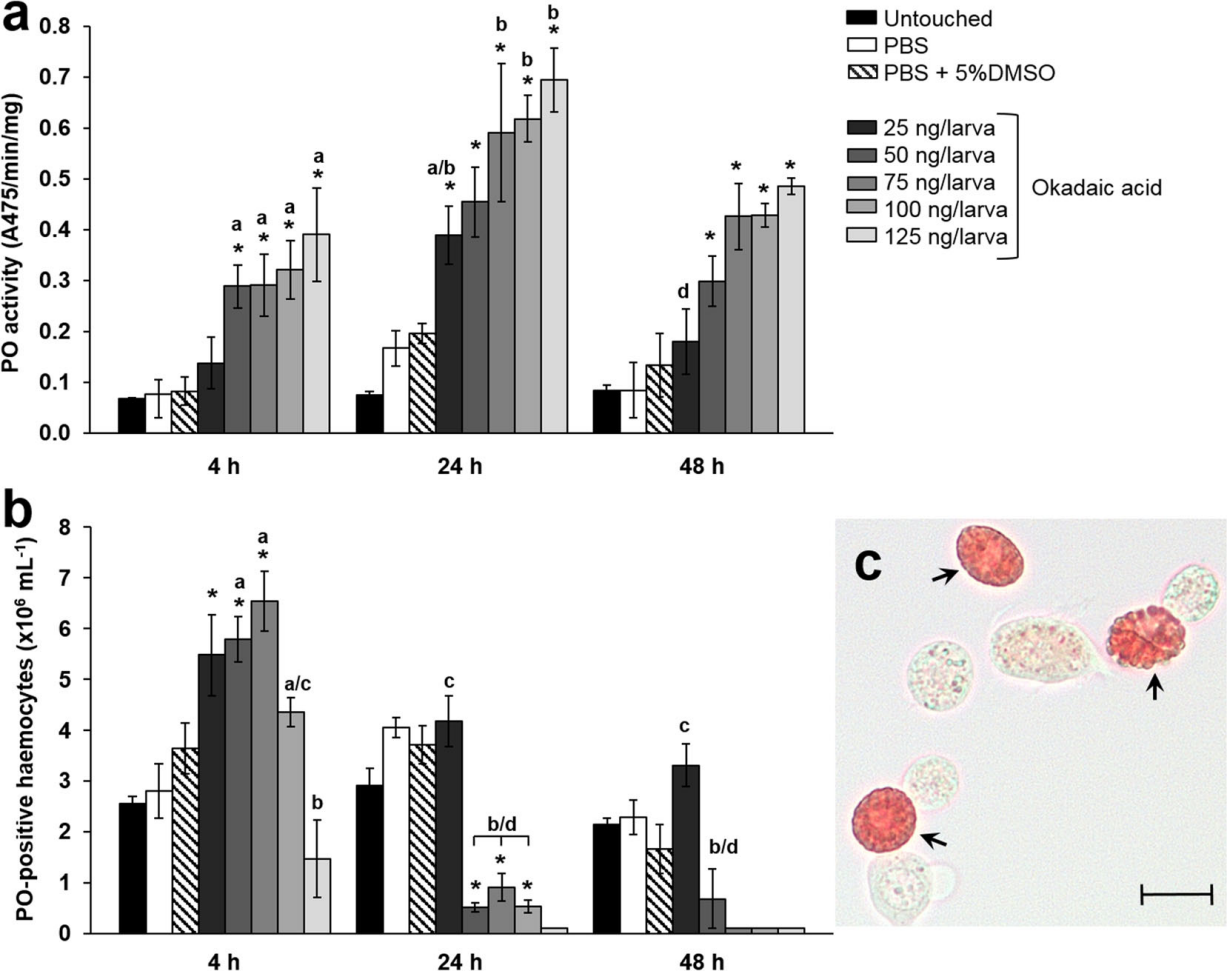
Phenoloxidase-associated activities in *Galleria mellonella* injected with increasing concentrations of okadaic acid, 0–125 ng/larva. **a** Phenoloxidase (PO) enzyme activities were recorded over a 48-h period. **b** The numbers of haemocytes staining positively for PO were recorded over a 48-h period. All values are represented by the mean ± SE (*n* = 18 per treatment, 144 in total). An asterisk indicates a significant difference (*P* < 0.05) relative to the PBS control. Unshared letters represent significant differences determined by Tukey’s multiple comparisons. **c** Image depicts haemocytes stained positively (black arrows) or unstained for the presence of PO. Scale bar represents 10 μm

Melanisation of the insect haemolymph is a key response to the presence of infectious agents (microbes, viruses, or parasites), toxins, or abiotic stressors (Whitten and Coates 2017). The early enzymatic steps that contribute to melanin formation are coordinated by phenoloxidases (POs), which are stored as inactive precursors (proPO) inside certain haemocytes. We observed significant increases in PO activity in the haemolymph of insects challenged with OA (Fig. 3a). PO activity increased in a dose-dependent manner (*F*(7, 48) = 29.46, *P* < 0.0001) and fluctuated over time (*F*(2, 48) = 24.95, *P* < 0.0001). Product formation (dopaminechrome) peaked at 24 h p.i. with 0.39–0.69 Abs/mg/min for OA-treated insects (25–125 ng/larva) compared to the controls (Fig. 3a). Although PO activities decreased by 48 h p.i. for all treatments, levels remained significantly higher for those insects exposed to ≥ 75 ng of the toxin. PO activities in control and untouched larvae followed the same pattern as OA-treated insects; however, activity remained lower at 0.067–0.196 Abs/mg/min.

We used a PO-specific cellular stain to further explore the effect(s) of OA on haemocytes. As concentrations of the toxin increased, a corresponding decrease in the number of PO-positive haemocytes was observed (Fig. 3b, c). Although both time (*F*(2, 48) = 100.8, *P* < 0.0001) and dose (*F*(7, 48) = 22.19, *P* < 0.0001) of OA significantly affected numbers of PO-positive haemocytes, it was time that accounted for the majority of variation observed, ~ 35%. The number of PO-positive haemocytes (Fig. 3b) correlated inversely to the increase in PO enzyme activities (Fig. 3a); therefore, we surmise that haemocytes are rupturing in the presence of OA and releasing PO into the haemolymph. Strikingly by 48 h p.i., < 0.01% of haemocytes stained positively for PO in larvae treated with ≥ 75 ng toxin, despite high levels of enzyme activity remaining detectable in the haemolymph. Conversely, the proportions of PO-positive haemocytes in the haemocoel of control and untouched larvae remained between 5.2 and 12.2% over the 48-h period (Supplementary Table 2).

### Toxicity of OA when administered using intrahaemocoelic injection versus force-feeding

**Fig. 4:**
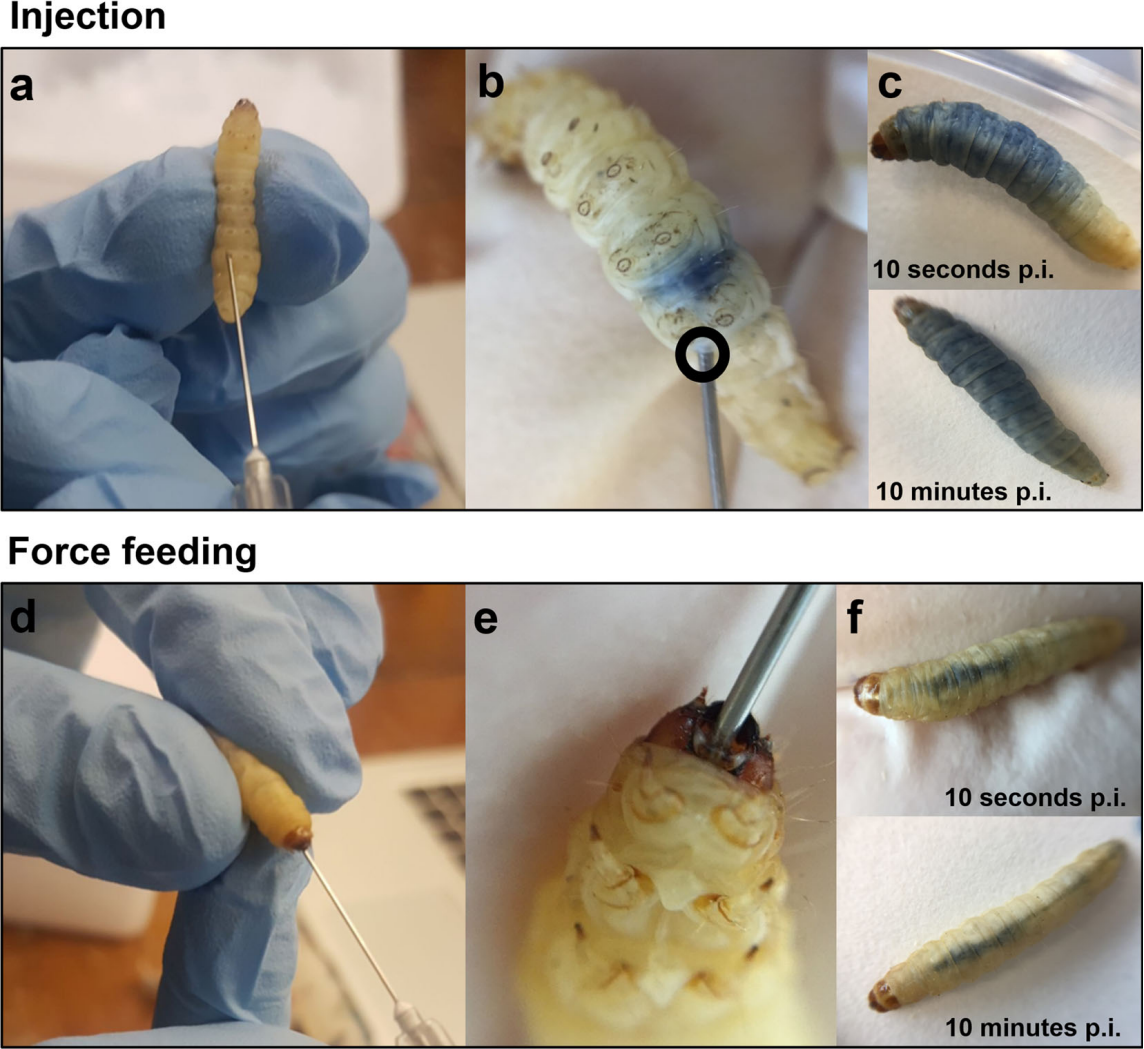
Administration of okadaic acid via intrahaemocoelic injection and force-feeding (gavage). Images **a**, **b**, **d** and **e** depict larvae of *Galleria mellonella* during inoculation, with **c** and **f** representing larvae 10 s and 10 min post-inoculation (p.i.). To illustrate the distribution of inoculum between the two methods, a solution of 0.4% [*w*/*v*] trypan blue in PBS pH 7.4 was used. The dye [20 μL/larva] remains in the insect gastrointestinal tract (mainly the midgut) when administered orally, whereas the dye disperses when injected directly into the haemocoel (body cavity)

**Fig. 5:**
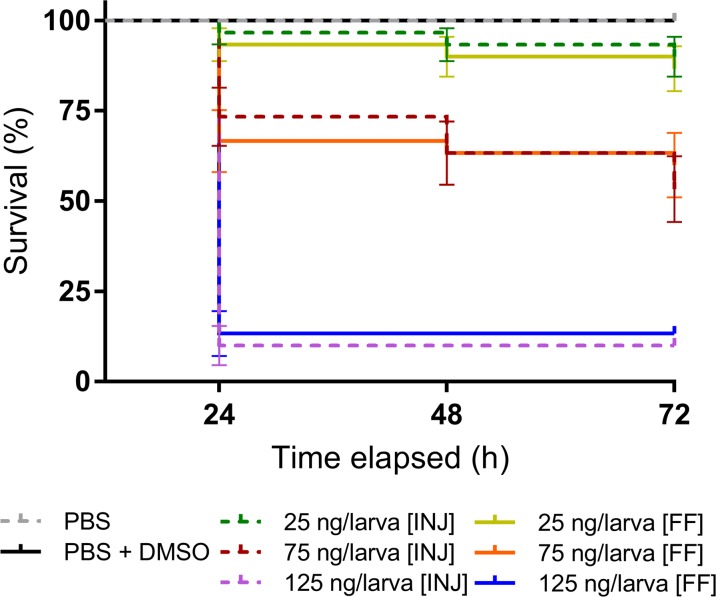
Okadaic acid intoxication of *Galleria mellonella* larvae via intrahaemocoelic injection [INJ] and force-feeding [FF]. Survival of *G*. *mellonella* after exposure to increasing concentrations of okadaic acid (25, 75, and 125 ng/larva). Post-inoculation, larvae were incubated at 30 °C in the dark and monitored over a 72-h period. Larvae unresponsive to touch were considered dead. Values are represented by the mean ± 95% CI (*n* = 30 per treatment, 300 in total). No mortalities occurred in insects through force-feeding or injection with 20 μL phosphate-buffered saline (PBS) or 20 μL PBS containing 5% dimethyl sulfoxide (DMSO)

Humans encounter OA when they consume contaminated shellfish; consequently, we investigated the relative toxicity of OA administered by force-feeding (gavage) compared to direct injection into the haemocoel (Fig. 4). We monitored the dissemination of tracer dye in insect larvae injected or force-fed a 20-μL inoculum. The inoculum dispersed immediately throughout the haemocoel (body cavity) when injected through the cuticle (Fig. 4a–c). Conversely, the inoculum remained within the insect gastrointestinal tract (i.e. the midgut) when force-fed and did not appear to leak into the surrounding haemolymph (Fig. 4d–f). Similar to the initial toxicity screening (Fig. 1a), the majority of insect mortalities occurred within 24 h regardless of the route of OA exposure (Fig. 5), up to 90% using the highest dose tested (125 ng/larva). LD_50_ values at 24 h p.i. were calculated as 252.8 μg/kg [*R*^2^ = 0.900, *n* = 30] for injected insects and 248.3 μg/kg [*R*^2^ = 0.947, *n* = 30] for those force-fed. In each case, OA had a significant negative effect on survival when compared to the controls (log-rank (Mantel-Cox) tests, *χ*^2^(4) = 103.6 and *χ*^2^(4) = 86.35, *P* < 0.0001 for injection and gavage, respectively). Using a three-way ANOVA, we determined the dose of okadaic acid to account for 83.4% of the variation (*F*(2, 24) = 70.4, *P* < 0.0001), with < 1% each due to time (*F*(1, 24) = 1.346, *P* = 0.2574), and importantly, inoculation method (*F*(1, 24) = 0.1495, *P* = 0.7024). No further mortalities were recorded in either force-fed or injected insects at 96 h.

**Fig. 6:**
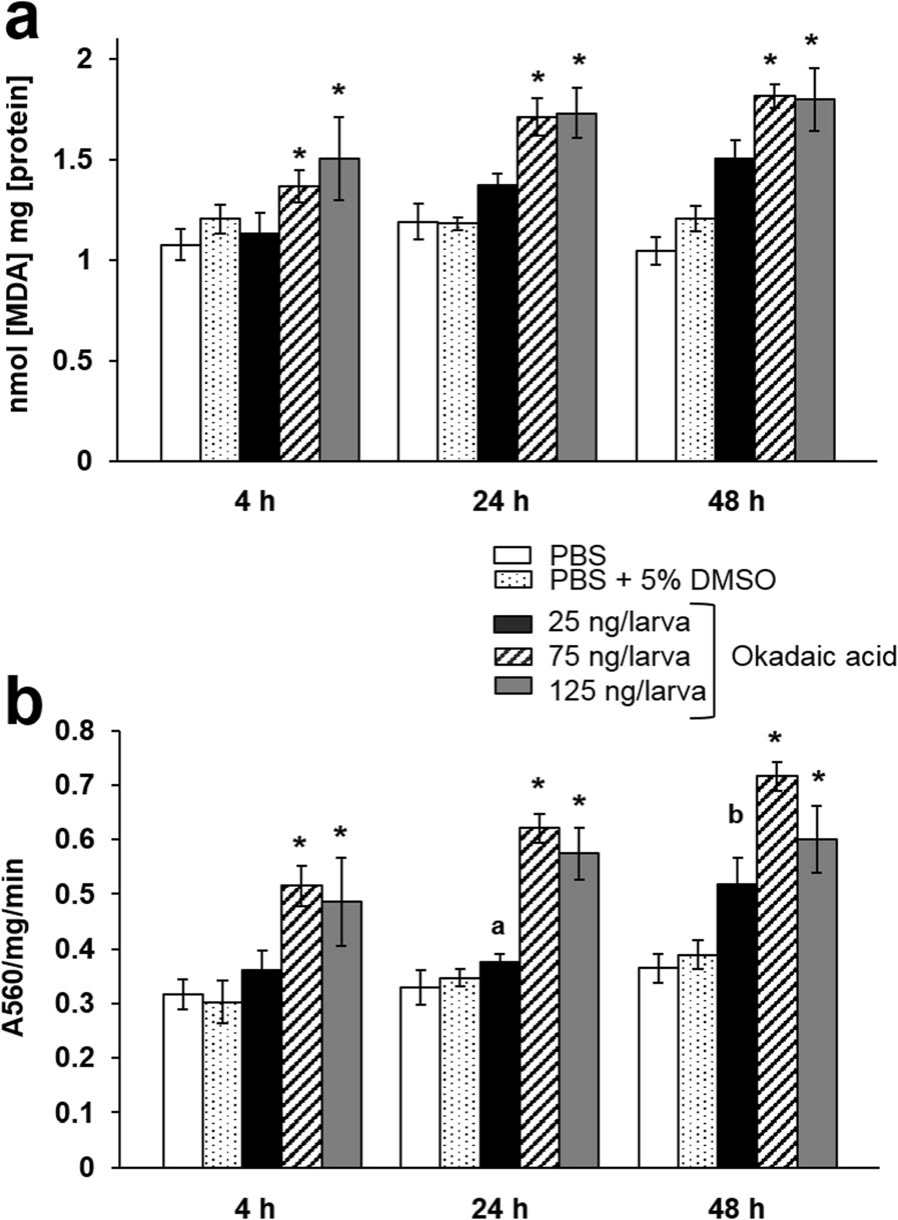
Oxidative damage in the midgut of *Galleria mellonella* when force-fed okadaic acid. **a** Lipid peroxidation was monitored by quantifying malondialdehyde, which is a by-product of oxidative damage. **b** Enzyme activity of the antioxidant, superoxide dismutase, was quantified over a 48-h period. An asterisk indicates a significant difference (*P* < 0.05) relative to the PBS control. Unshared letters between the 25-ng/larva treatment at 24 h and 48 h represent a significant difference (determined by Tukey’s multiple comparisons). All values are represented by the mean ± SE (*n* = 54 per treatment, 270 in total) for **a** and **b**. It should be noted that extracted midguts from six insects per treatment per time point were pooled

Okadaic acid disrupts gut homeostasis in rodent models leading to tissue damage, cell death, lipid peroxidation, and protein carbonylation (see Table 1). To see if force-feeding the toxin also induced similar symptoms in insect larvae, we measured the activity of the enzymatic antioxidant, superoxide dismutase (SOD), and levels of malondialdehyde (MDA; by-product of lipid peroxidation) in the midgut. OA treatment, particularly doses of 75 and 125 ng/larva, led to significant increases in MDA levels (*F*(4, 30) = 19.6, *P* < 0.0001; Fig. 6a) and SOD activities (*F*(4, 30) = 30.4, *P* < 0.0001; Fig. 6b) in the midgut when compared to the PBS control. At 48 h p.i., SOD activity peaked at 0.6–0.72 Abs/mg/min and MDA concentration reached 1.79–1.81 nmol/mg. No measurable oxidative damages were recorded in the midgut of insects fed PBS alone or PBS + 5%DMSO (Fig. 6).

**Fig. 7:**
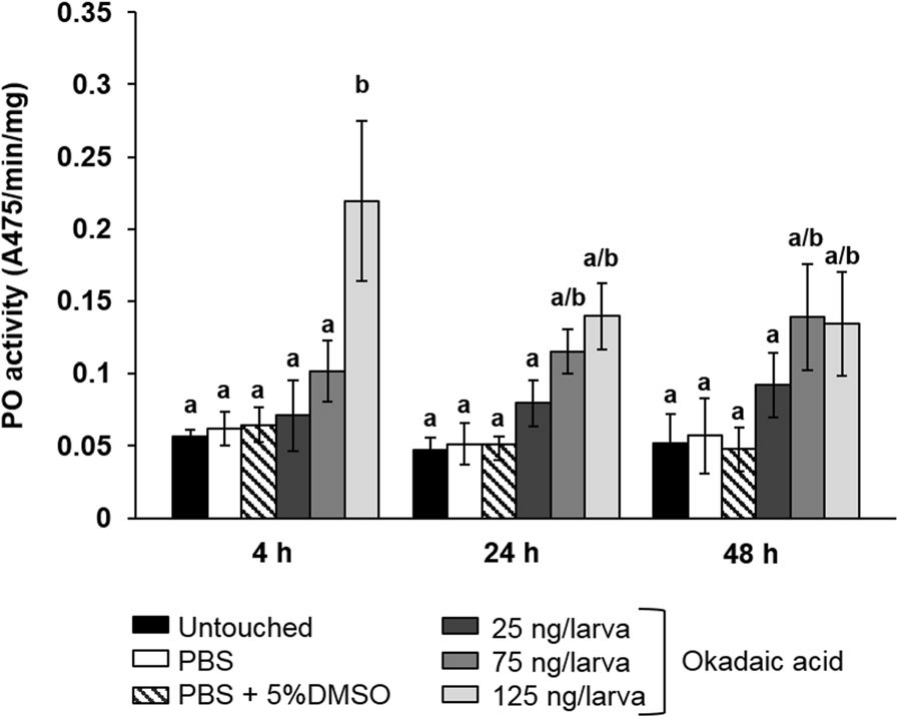
Phenoloxidase activity in the haemolymph of *Galleria mellonella* force-fed okadaic acid, 0–125 ng/larva. Phenoloxidase (PO) enzyme activities were recorded over a 48-h period. All values are represented by the mean ± SE (*n* = 18 per treatment, 108 in total). Unshared letters represent significant differences determined by Tukey’s multiple comparisons (*P* < 0.05)

Additionally, we monitored PO activities in the haemolymph of larvae force-fed OA (Fig. 7). Changes in PO activities were impacted significantly by dose (*F*(5, 36) = 14.47, *P* < 0.001) but not by time (*F*(2, 36) = 0.836, *P* = 0.441). Product formation (dopaminechrome) peaked at 4 h p.i. in larvae treated with 125 ng OA (0.219 Abs/mg/min) and remained > 0.115 Abs/mg/min for both 75 and 125 ng/larva at 24 and 48 h. PO activities in control and untouched larvae were consistent throughout the experimental period, 0.048–0.071 Abs/mg/min. Although we observed an increase in PO activities in response to oral doses of OA, the levels were not as high as those in insects directly injected with OA (Fig. 3a).

## Discussion

We present clear evidence that *G*. *mellonella* larvae exposed to OA have comparable LD_50_ values to rodent models (Fig. 1, Table 1) and display broad symptoms of immune-cytotoxicity (Figs. 2 and 3) and oxidative damage (Fig. 6). We observed little difference in lethality between force-feeding and injection (Fig. 5); therefore, either administration route would be suitable for screening purposes. Usually, ≥ 3-fold more OA is needed to induce lethality in rodents force-fed compared to intraperitoneal injection (Munday 2013; Vieira et al. 2013). There is great variation in the toxicity of OA in mice and rats across the literature, which is influenced not only by the route of inoculation (listed in Table 1), but also by the weight, strain, and sex of the experimental animals, parameters that are not always standardised (Suzuki 2012, 2014). A meta-analysis carried out by Botana et al. (2017) concluded that there is no correlation between LD_50_ values from intraperitoneal injection and force-feeding OA in vertebrate systems. We recorded differences in larval survival between the initial toxicity screen (Fig. 1, LD_50_ = 239.6 μg/kg) and our comparative study (Fig. 5, LD_50_ = ~ 252.8 μg/kg), which may be due to the alternative sources of insects used—commercial suppliers versus those reared in-house, respectively. Tsai et al. (2016) stated previously that batch-to-batch variability and environmental parameters (e.g. temperature, nutrients) might result in differential larval survivability between trials. This broad criticism concerning the reproducibility of *G*. *mellonella* as a model could be resolved with the availability of genetically tractable populations akin to drosophilid resources. We would also draw the reader’s attention to the likelihood of other endpoints being impacted because of background effects, e.g. immune gene expression, and should be considered carefully when selecting *G*. *mellonella*.

Few studies have focussed on the effects of OA on immunity. López et al. (2011) reported on the immune-regulatory properties of OA exposed to T-lymphocytes from the mouse cell line EL-4. Over a 72-h period, OA (≥ 50 nM) reduced T cell receptor complex expression, which is involved in cell sensitivity to antigens. Importantly, OA-induced immunomodulation was mediated by the inhibition of protein phosphatase 2A. OA is a well-characterised inhibitor of protein phosphatases 1 and 2A, which has long been considered the cause of DSP-related symptoms, yet Munday (2013) suggests OA may have several mechanisms of action facilitating its neurotoxicity, immunotoxicity, and genotoxicity. In excess of 100 nM, OA can induce apoptosis in human leukocytes in vitro and necrosis at 1000 nM (Valdiglesias et al. 2011). In our study, at the equivalent doses of 100 and 125 ng/larva (~ 124–155 nM OA), haemocyte counts reduced by 50% (Fig. 2a), and up to 40% of the remaining cells were dying/dead (Fig. 2b). Decreases in total haemocyte numbers followed a dose-dependent response, yet the highest proportion of dying/dead cells was observed using 75 ng/larva (Fig. 2). We speculate that OA at 75 ng/larva induces apoptotic cell death whereas higher doses are sufficient to induce necrosis or cytolysis. Trypan blue does not distinguish between early apoptotic (dying) cells and late apoptotic/necrotic (dead) cells. Okadaic acid also induced sufficient oxidative damage in the midgut that by 48 h SOD activities were elevated to assist in detoxification.

Considering shellfish immunity, Prado-Alvarez et al. (2013) exposed clam (*Ruditapes decussatus*) haemocytes to OA (10–500 nM) and recorded significant increases in cell death and reduced phagocytic capacity within 4 h in vitro. Mussel (*Mytilus galloprovincialis*) haemocytes incubated with OA over the same concentration range incurred DNA damage and were functionally compromised (Prego-Faraldo et al. 2015). A transcriptomic survey of OA-treated *M*. *galloprovincialis* identified 58 mRNAs encoding stress-associated proteins and cellular synthesis (Manfrin et al. 2010). The accumulation of OA in shellfish tissue appears to weaken the immune response, and consequently, they may be more susceptible to disease.

The mechanistic similarities between the innate immune systems of insects and mammals have led to the development of certain insects, e.g. *Drosophila melanogaster* and *G*. *mellonella*, as alternative in vivo models. These species are used successfully to reduce the reliance on vertebrates when assessing microbial and viral infectivity (Ramarao et al. 2012; Champion et al. 2016). Traditionally, microbes and/or toxins are administered by direct injection into the insect body cavity (haemocoel, Fig. 4), and this approach can discriminate between virulent versus non-virulent strains. Two previous studies attempted to develop cockroaches (*Nauphoeta cinerea*) and/or locusts (*Schistocerca gregaria*) as bioassays for the detection of saxitoxin—the causative agent of paralytic shellfish poisoning (Ruebhart et al. 2011 and Cook et al. 2006, respectively). The accuracy and/or reliability of this approach is unclear when we consider exposure to shellfish-poisoning toxins is by ingestion.

Maguire et al. (2016) assessed the relative toxicity of common food preservatives (e.g. sodium benzoate) using *G*. *mellonella* and found little difference between larvae that were injected or force-fed. Their calculated LD_50_, LD_80_, and IC_50_ values were in good agreement with data recorded for rats and cultured HEp-2 cells. The importance of this study is not to be overlooked. When characterising biocides, the route of exposure should be mimicked most closely—particularly when validating results obtained using substitutes to rodents and non-human primates, i.e. insect larvae. Over the last decade, the popularity of *G*. *mellonella* as a surrogate model of pathobiology and innate immunity has increased substantially and now includes marine pathogens that cause gastroenteritis when they encounter the human gut—*Vibrio* (*Listonella*) *anguillarum*, *V*. *cholerae*, and *V*. *parahaemolyticus* (McMillan et al. 2015; Bokhari et al. 2017; Wagley et al. 2018). The tissue structures and microbiome(s) of the insect gastrointestinal tract are similar to that of mammals (Mukherjee et al. 2013; Maguire et al. 2016, 2017). Many species of gut bacteria, including *Firmicutes and Clostridium*, are found on the microvilli of the human intestine and insect midgut (Sekirov et al. 2010; Spor et al. 2011; Dubovskiy et al. 2016). Normal gut profiles of microbes correlate broadly with disease resistance in humans, and in insects, the resident microbes are regulated by the innate immune system (Koch and Schmid-Hempel 2012). The utility of *G*. *mellonella* as a gut-specific model has not been investigated fully and offers much potential to explore the inextricable links between immunity, metabolism, and symbionts.

Although no cases of OA-induced mortality exist for humans, concentrations ≥ 75 ng/larva (≥ 242 μg/kg) resulted in > 65% reduction in *G*. *mellonella* survival, highlighting their possible use as a general screening tool for foodstuffs with suspected contamination of shellfish-poisoning toxins. Furthermore, larvae could be employed to calculate the lethality or cytotoxic potential of marine phytotoxins and their congeners (dinophysistoxin-2, azaspiracids, pectenotoxins, domoic acid, etc.). Identification of toxins still needs to be performed using specialised instruments such as mass spectrometry. Collectively, our evidence suggests OA is an immune-toxin, affecting blood cell (haemocyte) viability and functionality and leading to uncontrolled levels of PO activity, which generates highly reactive oxygen and nitrosative species as by-products. As PO levels peaked, we detected a corresponding decrease in haemocyte numbers. Even the sublethal dose of 25 ng/larva led to increases in immune cell death within 4 h. This dose also appeared to disrupt gut homeostasis—albeit the response was delayed compared to the higher doses tested, and not statistically significant when compared to the controls. Increasing the dose to 50 ng/larva reduced survival by 25% within 24 h. Interestingly, 50 ng/larva is equivalent to 161.3 μg/kg, which is the approximate upper regulatory limit for contaminated shellfish tissues (160 μg/kg) set by the Food Standards Agency UK (FSA 2018).

Our data demonstrates clearly the sensitivity of *G*. *mellonella* larvae to physiologically relevant quantities of OA. We have compared the toxicity of OA in insect larvae to rodent models using LD_50_ values as a general toxicology endpoint and, in doing so, validated the use of *G*. *mellonella* as a tool for studying DSP-associated marine toxins. By performing several immunotoxicological assays, we also posit that OA is a candidate immune-modulator and warrants further investigation.

## Electronic supplementary material

ESM 1 (DOCX 15 kb)

## Abbreviations

DSP: Diarrhetic shellfish poisoning
OA: Okadaic acid
PBS: Phosphate-buffered saline
MDA: Malondialdehyde
PO: Phenoloxidase
SDS: Sodium dodecyl sulphate
SOD: Superoxide dismutase
