# Supplementary material for: The insect, Galleria mellonella, is a compatible model for evaluating the toxicology of okadaic acid
Source: Cell Biol Toxicol. 2018 Nov 13;35(3):219–32. doi: 10.1007/s10565-018-09448-2 (PMC6556153; doi:10.1007/s10565-018-09448-2)
Supplement: Supplementary file 1 — (DOCX 15 kb) [file 10565_2018_9448_MOESM1_ESM.docx]

**Supplementary Table 1** Survival analyses of *Galleria mellonella* larvae injected with okadaic acid. Values represent outputs from pairwise comparisons (Log-rank [Mantel-Cox] tests) and Hazard Ratio(s) (Mantel-Haenszel) with 95% CI.

|  |  | Okadaic acid [ng/larva] | | | | |
| --- | --- | --- | --- | --- | --- | --- |
|  | PBS/DMSO | 25 | 50 | 75 | 100 | 125 |
| PBS | *X*^2^ (1) = 1.04, P = 0.3078  A/B = 0.505 (0.136-1.877) | *X*^2^ (1) = 3.006, P = 0.081  A/C = 0.259 (0.096-0.704) | *X*^2^ (1) = 20.32, **P < 0.0001**  A/D = 0.1599 (0.072-0.355) | *X*^2^ (1) = 59.64, **P < 0.0001**  A/E = 0.0743 (0.0384-0.144) | *X*^2^ (1) = 75.64, **P < 0.0001**  A/F = 0.0578 (0.0304-0.109) | *X*^2^ (1) = 101.1, **P < 0.0001**  A/G = 0.0413 (0.0221-0.0768) |
| PBS/DMSO |  | *X*^2^ (1) = 3.054, P = 0.0805  B/C = 0.4411 (0.176-1.105) | *X*^2^ (1) = 14.11, **P = 0.0002**  B/D = 0.2345 (0.11-0.499) | *X*^2^ (1) = 51.22, **P < 0.0001**  B/E = 0.0958 (0.0504-0.182) | *X*^2^ (1) = 67.04, **P < 0.0001**  B/F = 0.0725 (0.0386-0.1359) | *X*^2^ (1) = 92.04, **P < 0.0001**  B/G = 0.0503 (0.0272-0.0925) |
| 25 ng/larva |  |  | *X*^2^ (1) = 5.103, **P = 0.0239**  C/D = 0.468 (0.234-0.934) | *X*^2^ (1) = 33.48, **P < 0.0001**  C/E = 0.1637 (0.0887-0.302) | *X*^2^ (1) = 47.53, **P < 0.0001**  C/F = 0.1194 (0.0652-0.2184) | *X*^2^ (1) = 68.92, **P < 0.0001**  C/G = 0.0819 (0.0454-0.1479) |
| 50 ng/larva |  |  |  | *X*^2^ (1) = 14.21, **P = 0.0002**  D/E = 0.3212 (0.1779-0.579)**^1^** | *X*^2^ (1) = 24.05, **P < 0.0001**  D/F = 0.2304 (0.1282-0.4143) **^2^** | *X*^2^ (1) = 40.13, **P < 0.0001**  D/G = 0.1543 (0.0866-0.2751) **^3^** |
| 75 ng/larva |  |  |  |  | *X*^2^ (1) = 1.302, P = 0.254  E/F = 0.707 (0.3897-1.283)**^4^** | *X*^2^ (1) = 6.829, **P = 0.009**  E/G = 0.45 (0.2473-0.8191)^5^ |
| 100 ng/larva |  |  |  |  |  | *X*^2^ (1) = 1.998, **P = 0.1575**  F/G = 0.649 (0.3557-1.182)**^6^** |
| 125 ng/larva |  |  |  |  |  |  |

**1** [Reciprocal] Hazard Ratio = 3.11, 1.725 to 5.62

**2** [Reciprocal] Hazard Ratio = 4.34, 2.414 to 7.802

**3** [Reciprocal] Hazard Ratio = 6.48, 3.634 to 11.55

**4** [Reciprocal] Hazard Ratio = 1.42, 0.7796 to 2.566

**5** [Reciprocal] Hazard Ratio = 2.22, 1.221 to 4.044

**6** [Reciprocal] Hazard Ratio = 1.54, 0.8459 to 2.811

**Supplementary Table 2** Frequency (%) of phenoloxidase-positive cells within the total circulating haemocyte population in *Galleria mellonella* larvae injected with okadaic acid. Shaded blocks represent the highest values.

|  | Time post-inoculation | | |
| --- | --- | --- | --- |
|  | 4 hours | 24 hours | 48 hours |
|  |  |  |  |
| Untouched | 8.66 % | 8.72 % | 6.85 % |
| PBS | 7.94 % | 12.15 % | 7.15 % |
| PBS/DMSO | 11.23 % | 11.05 % | 5.15 % |
| 25 ng/larva | 16.81 % | 13.24 % | 12.27% |
| 50 ng/larva | 19.56 % | 1.93 % | 2.82 % |
| 75 ng/larva | 30.83 % | 6.11 % | <0.01% |
| 100 ng/larva | 21.59 % | 3.59 % | <0.01% |
| 125 ng/larva | 10.43 % | <0.01% | <0.01% |
|  |  |  |  |
